# Assessment of knowledge, attitude, and practice related to brucellosis among livestock farmers and meat handlers in Saudi Arabia

**DOI:** 10.3389/fvets.2024.1410330

**Published:** 2024-06-24

**Authors:** Mohammed H. Alghafeer, Ebrahim F. Aldhukair, Abdullah H. Alzahrani, Abdullah S. Alsaedi, Omar N. Almutairi, Abdulsalam A. Aloliky, Masaad Saeed Almutairi, Abrar K. Thabit

**Affiliations:** ^1^Faculty of Pharmacy, King Abdulaziz University, Jeddah, Saudi Arabia; ^2^College of Pharmacy, Qassim University, Buraidah, Saudi Arabia; ^3^Department of Pharmacy Practice, College of Pharmacy, Qassim University, Buraidah, Saudi Arabia; ^4^Department of Pharmacy Practice, Faculty of Pharmacy, King Abdulaziz University, Jeddah, Saudi Arabia

**Keywords:** brucellosis, livestock, farmers, infection control, zoonotic infection, Saudi Arabia

## Abstract

**Background:**

Brucellosis is a bacterial zoonotic infection that is endemic in Saudi Arabia and associated with clinical and economic impacts. Several studies from countries endemic for brucellosis evaluated the knowledge and attitude of livestock farmers regarding brucellosis. However, no such study was conducted in Saudi Arabia. This study aimed to evaluate the knowledge, attitude, and practice of livestock farmers and meat handlers in Saudi Arabia.

**Methods:**

This was a cross-sectional questionnaire-based study, where participants were interviewed in-person in Arabic in livestock markets between September–December 2023. Convenient sampling was utilized. The questionnaire included basic demographics and questions to assess the knowledge, attitude, and practice toward personal protection and protection of the animals from brucellosis. The questionnaire was adapted from a previously validated survey and included 59 questions. Providing at least one correct answer to a certain question indicated a good knowledge about this item or a safe practice. The participants were divided into: farmers (shepherds working for the animal owners), commercial animal owners (those who rent a stockyard in the livestock market and employ farmers to sell their animals), and private animal owners (owners of private farms from which they sell their animals).

**Results:**

545 participants were interviewed (*n* = 291 farmers, *n* = 118 commercial animal owners, *n* = 113 private animal owners, and *n* = 23 animal slaughterhouse workers). >90% have heard of brucellosis. Lack of education and short experience (<5 years) of working with livestock were negatively associated with good knowledge of brucellosis symptoms and transmission (OR, 0.30; 95%CI, 0.10–0.94; *p* = 0.038 and OR, 0.23; 95%CI, 0.08–0.62; *p* = 0.004, respectively). Taking sick animals to the veterinarian was reported by 61.2%, whereas 36.4% follow safe practices when disposing of aborted fetuses. While 34% consume raw milk, only 10% consume rare/medium-rare meat. 51.2% acknowledged the need for more information on brucellosis.

**Conclusion:**

This study revealed the need to augment the knowledge of people working with animals, particularly those with no school education and those with short work experience, via providing educational visits or materials or through veterinarians. This should help them identify human and animal brucellosis symptoms and increase the knowledge on how to protect oneself and animals from this disease.

## 1 Introduction

Brucellosis (i.e., Malta fever) is a bacterial zoonotic infection caused by *Brucella* spp. that is endemic in many regions, including the Middle East. Saudi Arabia is one of the middle eastern countries where brucellosis is endemic (1). A study from a single region in Saudi Arabia that included 690 animals found a 9.4% overall seroprevalence of brucellosis with the highest being reported in sheep (32/227; 14.1%), followed by goats (18/189; 9.5%) and camels (15/274; 5.5%) (2). Brucellosis is associated with significant clinical and economic impacts and is typically diagnosed via serology or culture (3, 4). In humans, brucellosis can be uncomplicated, which results in non-specific symptoms, such as fever, malaise, arthralgia, weight loss, dry cough, and gastrointestinal symptoms (3). If untreated, brucellosis can become complicated by disseminating to vital organs, namely the heart (brucella endocarditis) and the central nervous system (neurobrucellosis) (3). It can also infect the intervertebral disc resulting in spondylodiscitis. In contrast, brucellosis in animals can manifest in the form of abortion, weak calves, and decreased milk production (5).

The main modes of transmission of brucellosis to humans are through consumption of unpasteurized dairy products and through inhalation of airborne bacteria during direct interaction with livestock infected with *Brucella* spp., such as cattle, camels, goats, and sheep (3, 6). Livestock farmers are at risk of contracting the disease via airborne transmission, and be the reason for infection of other individuals from the public when selling unpasteurized dairy products that were milked from infected animals (3, 7). Lack of occupational hygiene and unsafe handling of infected live or dead animals can also increase risk of exposure to the disease for both the farmers and slaughterhouse workers (3, 7). Safeguarding the public from brucellosis starts by preventing it in the animal source. A key strategy for achieving this is vaccination of the animals as no human vaccine is currently available (8). Alternatively, when a vaccine is not accessible or unavailable, animal farmers should educate themselves on recognizing brucellosis symptoms in infected animals. Prompt treatment and avoiding milking such animals are essential. Additionally, the public should refrain from consuming unpasteurized dairy products from local farms or ensure they are properly boiled before consumption.

Four studies from different regions in Saudi Arabia surveyed the public regarding their knowledge and attitude regarding brucellosis (9–12). Collectively, the studies included 3,353 participants. Having heard of brucellosis was reported by a total of 1,913 (57.1%) participants with a range of 50–73.6% between the studies who mostly demonstrated a good knowledge (range 53.1–90%). Factors such as being a male, old, and having a high level of education were significantly associated with good knowledge (10–12). While evaluating the understanding of the general public regarding brucellosis holds significance, it is crucial to assess the knowledge and behavior of individuals working with animals. These frontline workers (livestock farmers and slaughterhouse workers) face direct exposure to the disease, making their awareness and practices of utmost importance.

Several studies in the literature from different countries endemic for brucellosis evaluated the knowledge, attitude, and practices of livestock farmers regarding brucellosis (13–16). However, no similar study was conducted in Saudi Arabia on such a population as well as slaughterhouse workers who are typically prone to contract brucellosis. Therefore, this study aimed to evaluate the knowledge, attitude, and practice of livestock farmers and animal slaughterhouse workers in various regions in Saudi Arabia, where brucellosis was reported to be mostly prevalent in the literature (1). Results from this study would be shared with the relevant authorities in Saudi Arabia along with appropriate recommendations to reduce the impact of brucellosis in the country.

## 2 Methods

### 2.1 Study design and population

A cross-sectional questionnaire-based study was conducted between September and December 2023 in Saudi Arabia. The study involved in-person interviews in Arabic. Eligible participants were livestock farmers, animal owners, and slaughterhouse workers aged ≥16 years regardless of experience duration and animal species. Both Saudi citizens and residents were included. As such, participants who were temporarily visiting from neighboring countries to sell their livestock in Saudi markets were excluded. The study was approved by the Research Ethics Committee of the Faculty of Pharmacy, King Abdulaziz University, Jeddah, Saudi Arabia (Ref. PH-1444-42).

Saudi Arabia is divided into 13 administrative regions that are collectively divided into five major regions (central, western, eastern, northern, and southern). The study investigators made trips to meet the farmers and animal owners in-person, where they visited the major livestock markets and various local farms in one or more major cities in each of the five regions. The participants were approached where they work and sell their products, including livestock markets and local farms. Convenience sampling method was employed, where the investigators randomly approached potential participants and interviewed those who were comfortable to take part in the study. One slaughterhouse in the western region was visited to collect responses to relevant questions from its workers. Overall, recruitment of participants continued until all the visits were concluded even when the required sample size was achieved.

### 2.2 Sample size calculation

A sample size of 385 participants was needed to meet a confidence level of 95% and a margin of error of 5% based on an estimated population size of 100,000 livestock farmers and animal owners in Saudi Arabia. The population size was estimated based on the latest available statistics on the number of animal farms in Saudi Arabia, which showed a total of 61,663 animal farms in 2015 (17). The population size used in the calculation was selected based on an assumption that the number of animal farms as well as the number of farmers and animal owners have increased over the years until this study was conducted. Additionally, this selected population size represents the upper limit, as any larger population would yield the same sample size result. Sample size calculation was done using Qualtrics® sample size calculator (18).

### 2.3 Method of data collection

The questions of the questionnaire were adapted from an already validated questionnaire from a similar previously published study (13). Some questions were omitted and/or edited for cultural appropriation purposes. It involved collection of demographic information (10 questions), followed by assessment of knowledge about brucellosis (8 questions), information about the livestock owned or managed by the farmer (17 questions), as well as assessment of attitude and practice toward potentially infected animals, consumption of raw dairy products, and occupational hygiene practices (24 questions). A Google form was created into which the responses were entered by the study investigators. The questionnaire was initially piloted with three farmers before commencing the study at a large scale to ensure that the translation to Arabic was appropriate and that the questions were understandable and culturally appropriate. Good knowledge and safe practice were considered if the participants provided at least one correct answer to the respective questions. A copy of the questionnaire is available in the Supplementary material.

### 2.4 Statistical analysis

Upon completion of responses collection, data were coded in the resultant spreadsheet on Microsoft Excel version 2,404 (Microsoft Corp., Seattle, WA, United States) and then analyzed using SPSS version 24.0 (IBM Corp., Armonk, NY, United States). The participants were grouped depending on their job into farmers (shepherds working for the owners of the animals), commercial animal owners (those who rent a stockyard in the livestock market and employ farmers to sell their animals), and private animal owners (those who have their own private farms from which they sell their animals and do not use the public livestock markets). Data were compared using Chi-square for categorical variables and Kruskal-Wallis for continuous variables. Multivariable logistic regression was utilized to evaluate the association of different factors/independent variables (namely age group, region, educational level, job type, and work experience level) with the dependent (outcome) variables of good knowledge and safe occupational practices to protect self and animals from brucellosis by calculating odds ratios (OR) and 95% confidence intervals (95% CI). Each question was analyzed individually for good knowledge and safe practice with the mentioned factors. Statistical significance was determined by a *p* value of <0.05.

## 3 Results

### 3.1 Demographics of farmers and animal owners and their animal husbandry practices

A total of 522 farmers and animal owners were interviewed, where 291 (55.6%) were farmers, 118 (22.7%) were commercial animal owners, and 113 (21.7%) were private animal owners. Most of the participants were from the central (*n* = 178; 34%) and western (*n* = 174; 33.3%) regions. More than half of the participants were in the age group of 31–50 years (*n* = 294; 56.32%). Many of the participants were non-Saudis (*n* = 324; 62%), mostly from Sudan (*n* = 255; 48.9%). In terms of education, one third of the farmers (*n* = 103/291; 35.4%) lacked formal school education. Conversely, almost half of the private animal owners held a college degree (*n* = 54/113; 47.8%). Further details on the participants’ characteristics are listed in Table 1. Moreover, information on animal, water, and fodder sources is shown in Supplementary Table S1.

**Table 1:** Demographics of included farmers and animal owners in Saudi Arabia (*n* = 522).

Characteristic	Farmers (*n* = 291)	Commercial owners (*n* = 118)	Private owners (*n* = 113)	*p* value
Age (years)	34 [27–43]	40 [29–51]	42 [37–49]	< 0.001
Age group (years)				< 0.001
16–17	4 (1.4)	3 (2.5)	0 (0)	
18–30	108 (37.1)	30 (25.4)	8 (7.1)	
31–50	152 (52.2)	55 (46.6)	87 (77)	
> 50	27 (9.3)	30 (25.4)	18 (15.9)	
Nationality				< 0.001
Saudi	1 (0.3)	86 (72.9)	111 (98.2)	
Non-Saudi	290 (99.7)	32 (27.1)	2 (1.8)	
Region				< 0.001
Western	136 (46.7)	4 (3.4)	34 (30.1)	
Central	77 (26.5)	56 (47.5)	45 (39.8)	
Southern	14 (4.8)	32 (27.1)	18 (15.9)	
Northern	22 (7.6)	26 (22)	11 (9.7)	
Eastern	42 (14.4)	0 (0)	5 (4.4)	
Education				< 0.001
None	103 (35.4)	20 (16.9)	1 (0.9)	
Elementary school	68 (23.4)	11 (9.3)	4 (3.5)	
Middle school	54 (18.6)	24 (20.3)	9 (8)	
High school	54 (18.6)	33 (28)	27 (23.9)	
Vocational college	5 (1.7)	11 (9.3)	18 (15.9)	
College/University	7 (2.4)	19 (16.1)	54 (47.8)	
Work experience (years)	10 [5–15]	10 [5.75–23]	13 [7.5–20]	< 0.001
Heard of brucellosis	266 (91.4)	113 (95.8)	111 (98.2)	0.023
Source of information				< 0.001
Friend/family	220 (75.6)	84 (71.2)	54 (52.2)	
Social media	10 (3.4)	8 (6.8)	43 (38.1)	
TV/Radio/Newspaper	10 (3.4)	3 (2.5)	15 (13.3)	
Healthcare worker	32 (11)	27 (22.9)	35 (31)	
Previous infection	8 (2.7)	6 (5.1)	12 (10.6)	
Experience	4 (1.4)	1 (0.8)	3 (2.7)	

Data are presented as *n* (%) or median [interquartile range].

Having experienced at least three of the constitutional symptoms of brucellosis, such as fever, night sweats, arthralgia, or myalgia within the last year was reported by a total of 120 of 522 participants (23%). Having any of these symptoms was reported by 37.3% (*n* = 28/75) of the participants who were ≥ 50 years, 20.7% (*n* = 61/294) of the participants who were 31–50 years old, and 19.9% (*n* = 29/146) of the participants who were 18–30 years old (*p* = 0.015). Most of those participants who had these symptoms came from the central region (*n* = 66/178; 37.1%), followed by the northern (*n* = 14/59; 23.7%) and the eastern (*n* = 10/47; 21.3%) regions (*p* < 0.0001).

### 3.2 Knowledge of farmers and animal owners about brucellosis

Table 2 shows the answers to the questions used to assess the participants’ knowledge of brucellosis. Most private and commercial owners were aware that *Brucella* can infect humans (96.5 and 97.5%, respectively), whereas 85.6% of the farmers were aware of that (*p* < 0.01). Regarding the transmission of *Brucella* to humans, private owners had the most correct answers, followed by commercial owners and farmers. A similar pattern was seen concerning the knowledge of *Brucella* transmission between animals; though, most farmers (41.6%) expressed their lack of knowledge. In terms of brucellosis symptoms in humans, fever was the most reported symptom, followed by arthralgia, lethargy/fatigue, headache, and back pain. It was noted that private owners were more knowledgeable than commercial owners and farmers (86.7% vs. 74.6 and 45.9%, respectively; *p* < 0.001). With regards to the level of knowledge on brucellosis symptoms in animals, farmers were the most to acknowledge lack of knowledge (54.3%) compared to commercial owners and private owners (39.8 and 11.5%, respectively; *p* < 0.01). Miscarriage was the most reported brucellosis symptom in animals, where 61.9% of private owners linked miscarriage to the disease compared to a smaller percentage of commercial owners (27.9%) and farmers (6.5%) (*p* < 0.001).

**Table 2:** Survey results on the knowledge of brucellosis among farmers and animal owners in Saudi Arabia (*n* = 522).

Item	Farmers (*n* = 291)	Commercial owners (*n* = 118)	Private owners (*n* = 113)	*p* value
*Brucella* can infect humans	249 (85.6)	115 (97.5)	109 (96.5)	< 0.001
How is *Brucella* transmitted to humans?				
Dairy products	130 (44.7)	77 (65.3)	99 (87.6)	< 0.001
Contact with blood or raw meat	98 (33.7)	57 (48.3)	85 (75.2)	< 0.001
Inhalation	96 (33)	49 (41.5)	37 (32.7)	0.225
Touching of animals	57 (19.6)	23 (19.5)	19 (16.8)	< 0.805
Raw meat consumption	53 (18.2)	41 (34.7)	79 (69.9)	< 0.001
I do not know	62 (21.3)	19 (16.1)	7 (6.2)	0.001
How is *Brucella* transmitted between animals?				
Inhalation	65 (22.3)	17 (14.4)	6 (5.3)	< 0.001
Direct contact	48 (16.5)	13 (11)	5 (4.4)	0.004
Consumption of contaminated food or water	29 (10)	24 (20.3)	15 (13.3)	0.018
Sexual contact	15 (5.2)	32 (27.1)	85 (75.2)	< 0.001
Unclean farms	15 (5.2)	7 (5.9)	1 (0.9)	0.113
Contact with infected blood	4 (1.4)	1 (0.8)	2 (1.8)	0.828
I do not know	121 (41.6)	37 (31.4)	9 (8)	< 0.001
What are the symptoms of brucellosis in humans?				
Fever	133 (45.9)	88 (74.6)	98 (86.7)	< 0.001
Arthralgia	78 (26.8)	38 (32.2)	47 (41.6)	0.015
Lethargy/fatigue	64 (22)	19 (16.1)	26 (23)	0.340
Headache	33 (11.3)	23 (19.5)	6 (5.3)	0.004
Back pain	33 (11.3)	18 (15.3)	6 (5.3)	0.050
Myalgia	9 (3.1)	3 (2.5)	2 (1.8)	0.757
Sweating	6 (2.1)	12 (10.2)	13 (11.5)	< 0.001
I do not know	87 (29.9)	17 (14.4)	7 (6.2)	< 0.001
What are the symptoms of brucellosis in animals?				
Fatigue	54 (18.6)	20 (16.9)	10 (8.8)	0.056
Fever	28 (9.6)	14 (11.9)	16 (14.2)	0.410
Loss of appetite	20 (6.9)	8 (6.8)	2 (1.8)	0.122
Miscarriage	19 (6.5)	35 (29.7)	70 (61.9)	< 0.001
Testicular swelling	18 (6.2)	21 (17.8)	34 (30.1)	< 0.001
Cold symptoms	17 (5.8)	1 (0.8)	0 (0)	0.003
Eye tears	13 (4.5)	4 (3.4)	0 (0)	0.076
Difficulty breathing	1 (0.3)	1 (0.8)	0 (0)	0.573
Diarrhea	1 (0.3)	0 (0)	0(0)	0.672
I do not know	158 (54.3)	47 (39.8)	13 (11.5)	< 0.001
Brucellosis is treatable in humans	243 (83.5)	112 (94.9)	88 (77.9)	< 0.001
How can brucellosis be prevented in humans?				
Occupational hygiene	106 (36.4)	51 (43.2)	57 (50.4)	0.031
Isolate the infected individual (animal or human)	36 (12.4)	11 (9.3)	5 (4.4)	0.055
Avoid raw milk consumption	21 (7.2)	38 (32.2)	52 (46)	< 0.001
Vaccine	19 (6.5)	3 (2.5)	1 (0.9)	0.025
Avoid raw meat consumption	6 (2.1)	7 (5.9)	19 (16.8)	< 0.001
Animal testing	2 (0.7)	0 (0)	2 (1.8)	0.296
I do not know	115 (39.5)	29 (24.6)	27 (23.9)	0.001
How can brucellosis be prevented in animals?				
Isolation of infected animals	82 (28.2)	33 (28)	23 (20.4)	0.253
Vaccination	60 (20.6)	18 (15.3)	20 (17.7)	0.429
Regular visits to the veterinarian	15 (5.2)	6 (5.1)	2 (1.8)	0.175
Good hygiene	11 (3.8)	12 (10.2)	7 (6.2)	0.041
I do not know	88 (30.2)	29 (24.6)	5 (4.4)	< 0.001

Data are presented as *n* (%).

**Figure 1:**
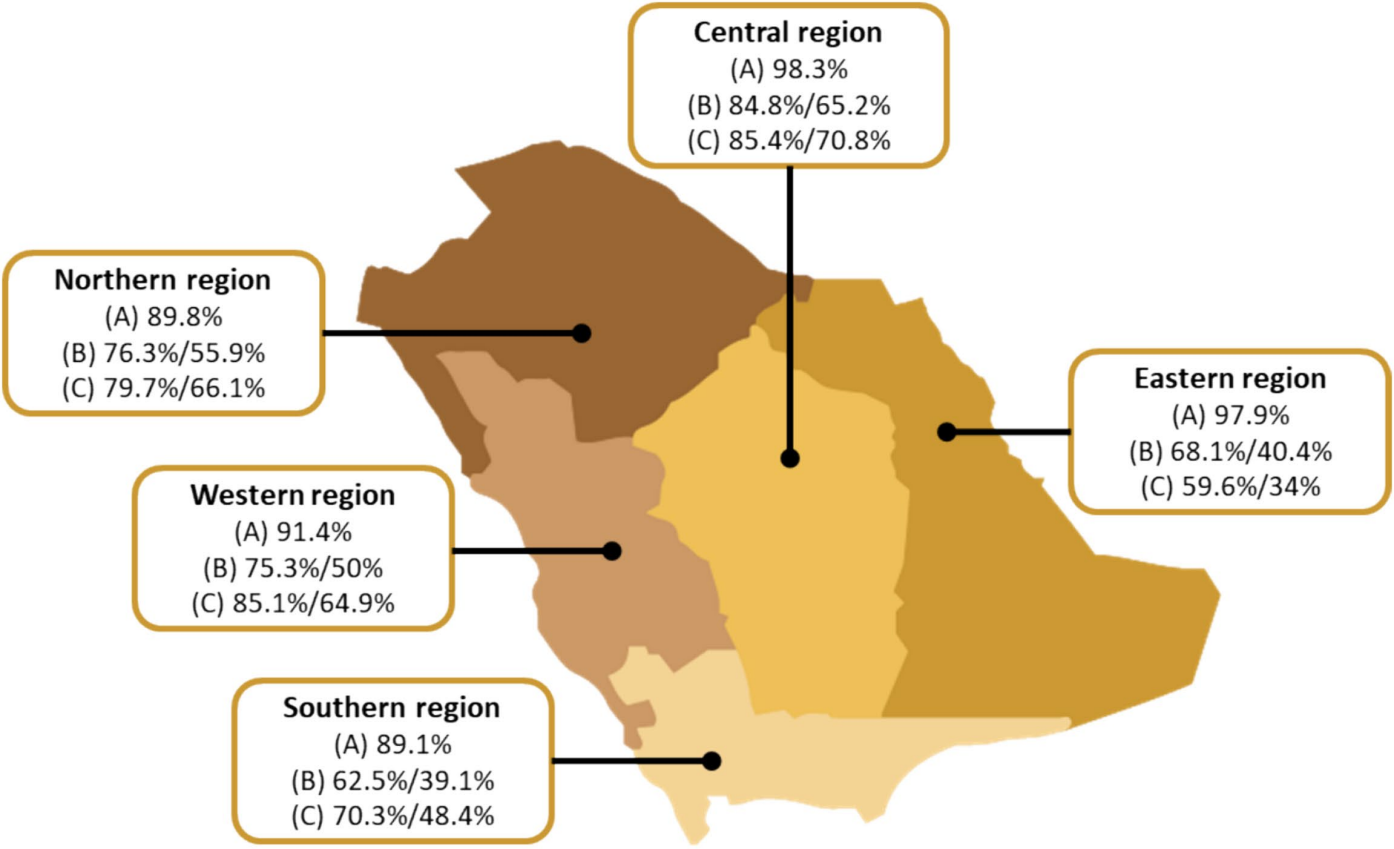
Level of knowledge of brucellosis as demonstrated by participants from different regions of Saudi Arabia the map depicts the percentages of participants who (A) have heard of brucellosis, (B) knew at least one correct symptom of brucellosis in humans/animals, and (C) knew at least one correct mode of transmission of brucellosis to humans/animals.

Most participants agreed that brucellosis is treatable in humans. In terms of the strategies to prevent brucellosis in humans, 39.5% of farmers were unaware of such strategies compared to commercial owners or private owners (24.6, 23.9%, respectively; *p* < 0.01). Similarly, farmers were less aware about brucellosis prevention in animals compared to commercial and private owners who stated lack of such knowledge (30.2, 24.6, and 4.4%, respectively; *p* < 0.01). Figure 1 shows the level of knowledge demonstrated by participants from different regions of Saudi Arabia.

### 3.3 Factors associated with good knowledge about brucellosis by farmers and animal owners

Several factors were evaluated in association of good knowledge of brucellosis in terms of its symptoms and modes of transmission. Lack of school education and an experience of working with livestock for <5 years were negatively associated with good knowledge of modes of brucellosis transmission to humans (i.e., selecting at least one correct answer out of 5 questions) (OR, 0.30; 95% CI, 0.10–0.94; *p* = 0.038 and OR, 0.23; 95% CI, 0.08–0.62; *p* = 0.004, respectively). A similar pattern of negative association was also seen with knowledge of modes of brucellosis transmission among animals, though with all the educational levels, being a farmer or a commercial owner, and a short experience of <5 years (6 questions; OR, < 1 for all the mentioned factors; *p* < 0.05). On the other hand, participants from the western and central regions demonstrated a better knowledge regarding brucellosis transmission among animals (OR, 2.47; 95% CI, 1.11–5.51; *p* = 0.027 and OR, 2.81; 95% CI, 1.21–6.53; *p* = 0.016, respectively). Additionally, lack of education, elementary school education, middle school education, high school, being from the southern region, and short work experience were negatively associated with good knowledge of brucellosis symptoms in humans (7 questions; OR, < 1 for all the mentioned factors; *p* < 0.05). Moreover, all the educational levels (except elementary), being a farmer or a commercial owner, and a short work experience, were negatively associated with good knowledge of brucellosis symptoms in animals (9 questions; OR, < 1 for all the mentioned factors; *p* < 0.05).

Participants from the central region, as well as commercial owners had higher odds of answering that brucellosis is treatable in humans (OR, 68.21; 95% CI, 5.44–854.77; *p* = 0.001 and OR, 5.70; 95% CI, 1.17–27.77; *p* = 0.031, respectively). Lack of education and education up to high school and being a farmer were associated with lower odds of knowing how to prevent brucellosis in humans (OR, < 1; *p* < 0.05). Conversely, participants from the northern regions were more likely to correctly answer the question pertaining to the prevention of brucellosis transmission to humans (OR, 15.14; 95% CI, 1.61–142.06; *p* = 0.017). Regarding the knowledge of how to prevent brucellosis from spreading among animals, only being a farmer or a commercial owner were significantly associated with correct answers (OR, 2.09; 95% CI, 1.12–3.93; *p* = 0.022 and OR, 1.87; 95% CI, 1.02–3.46; *p* = 0.044). Nonetheless, those with a short experience had lower odds of knowing how to prevent brucellosis in animals (OR, 0.30; 95% CI, 0.15–0.60; *p* = 0.001). Detailed results of the regression analyses of factors associated with different knowledge items are provided in Supplementary Table S2.

### 3.4 Attitude and practice of farmers and animal owners toward brucellosis control and prevention

**Figure 2:**
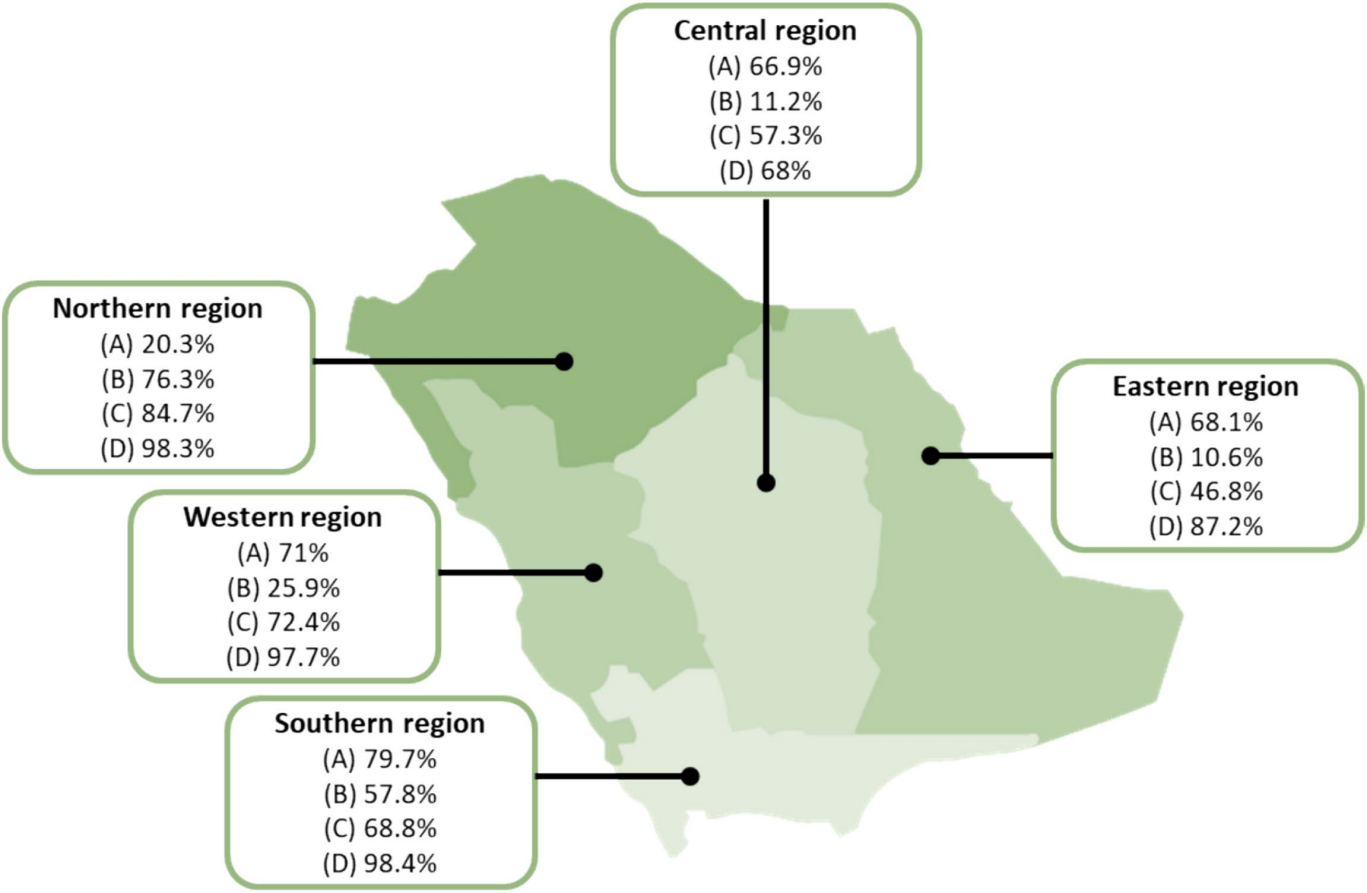
Attitude of participants from different regions of Saudi Arabia toward brucellosis control and prevention the map depicts the percentages of participants who (A) correctly manages a sick animal by taking it to the veterinarian, (B) correctly dispose of aborted fetus and placenta (by taking it to the veterinarian, burying it, or burning it), (C) do not consume raw dairy products, and (D) do not consume rare/medium-rare cooked meat.

Table 3 lists the responses to the questions pertinent to the participants’ attitude. In terms of vaccination against any disease, 159/291 (54.6%) of the farmers indicated that they do not vaccinate their livestock compared to 26/118 (22%) of commercial owners and 18/113 (15.9%) of the private owners (*p* < 0.0001). Further analysis showed that 23/113 (20.4%) of the private owners vaccinate their livestock against brucellosis compared to only 14/118 (11.9%) of the commercial owners and 18/291 (6.2%) of the farmers (*p* < 0.01). While more than two-thirds of the private owners (67.3%) stated that abortion was very serious and dangerous, most farmers (62.2%) did not see it as dangerous (*p* < 0.01) (Table 3). Almost one third of the private owners, commercial owners, and farmers consume raw milk. Nonetheless, most farmers, commercial and private owners (70.1, 71.2, and 92%, respectively; *p* < 0.01) believe that raw milk should be boiled before consumption. Figure 2 shows the attitude of the participants toward brucellosis control and prevention based on their region.

**Table 3:** Survey results on the attitude and practice toward brucellosis control and prevention among farmers and animal owners in Saudi Arabia (*n* = 522).

Item	Farmers (*n* = 291)	Commercial owners (*n* = 118)	Private owners (*n* = 113)	*p* value
I vaccinate my animals in general	132 (45.4)	92 (78)	95 (84.1)	< 0.001
I vaccinate my animals against *Brucella*	18 (6.2)	14 (11.9)	23 (20.4)	< 0.001
What *Brucella* vaccine do you use				< 0.001
I do not know	36 (12.4)	29 (24.6)	24 (21.2)	
Not available/do not use	251 (86.3)	83 (70.3)	75 (66.4)	
Ministry of Agriculture	0 (0.0)	1 (0.8)	7 (6.2)	
Pharmacy	4 (1.4)	5 (4.2)	7 (6.2)	
Did you receive an order from the MOH to vaccinate against Brucellosis?	60 (20.6)	17 (14.4)	16 (14.2)	0.171
Animals tested positive for brucellosis last year	15 (5.2)	12 (10.2)	26 (23.0)	< 0.001
Who diagnosed the animal?				< 0.001
Veterinarian	5 (1.7)	8 (6.8)	13 (11.5)	
Self/worker	4 (1.4)	2 (1.7)	6 (5.3)	
Ministry of Agriculture	0 (0.0)	1 (0.8)	4 (3.5)	
I do not know	6 (2.1)	0 (0.0)	1 (0.9)	
Not applicable	276 (94.8)	107 (90.7)	89 (78.8)	
What happened to the brucellosis animal?				< 0.001
Treated	6 (2.1)	6 (5.1)	6 (3.4)	
Died	4 (1.4)	1 (0.8)	1 (1.1)	
Slaughtered/disposed off	0 (0.0)	5 (4.2)	10 (2.9)	
Miscarriage/fatigue	1 (0.3)	0 (0.0)	5 (1.1)	
Nothing	0 (0.0)	0 (0.0)	3 (0.6)	
Not applicable	280 (96.2)	106 (89.8)	88 (90.8)	
How dangerous is abortion?				< 0.001
Not dangerous	181 (62.2)	26 (22.0)	10 (8.8)	
Serious	33 (22.7)	43 (36.4)	27 (23.9)	
Very serious	44 (15.1)	49 (41.5)	76 (67.3)	
How to handle diseased animal?				< 0.001
Treat by myself	60 (20.6)	37 (31.4)	48 (42.5)	
Veterinarian	220 (75.6)	78 (66.1)	53 (46.9)	
Slaughter & benefit from meat	5 (1.7)	3 (2.5)	8 (7.1)	
Get rid of it	0 (0.0)	0 (0.0)	4 (3.5)	
Not applicable	6 (2.1)	0 (0.0)	0 (0.0)	
How do you milk your animals?				< 0.001
By hand	69 (23.7)	68 (57.6)	87 (77.0)	
Machine	0 (0.0)	1 (0.8)	1 (0.9)	
I do not milk animals	222 (76.3)	49 (41.5)	25 (22.1)	
Who milks the animal?				< 0.001
Shepherd	68 (23.4)	56 (47.5)	71 (62.8)	
Owner	0 (0.0)	11 (9.3)	16 (14.2)	
Not applicable	223 (76.6)	51 (43.2)	26 (23)	
Do you wash your hands after milking?				< 0.001
Yes	84 (28.9)	81 (68.6)	82 (72.6)	
Not applicable	192 (66.0)	26 (22.0)	24 (21.2)	
Do you touch the placenta or aborted fetuses by your hand?	52 (17.9)	39 (33.1)	25 (22.1)	0.004
How do you manage aborted fetuses and placenta in cattle?				
Leave it	10 (3.4)	0 (0)	6 (5.3)	0.056
Feed it to dogs	12 (4.1)	6 (5.1)	19 (16.8)	< 0.001
Throw it in the trash	200 (68.7)	83 (70.3)	47 (41.6)	< 0.001
Take it to vet	9 (3.1)	5 (4.2)	1 (0.9)	0.296
Burn it	5 (1.7)	1 (0.8)	5 (4.4)	0.131
Bury it	48 (16.5)	21 (17.8)	30 (26.5)	0.064
Not applicable	9 (3.1)	5 (4.2)	0 (0)	0.111
What will you do if you suspect that an animal has brucellosis?				
Isolation	161 (55.3)	51 (43.2)	59 (52.2)	0.085
Treat at vet	122 (41.9)	69 (58.5)	23 (20.4)	< 0.001
Test	7 (2.4)	21 (17.8)	41 (36.3)	< 0.001
Slaughter	5 (1.7)	1 (0.8)	3 (2.7)	0.573
Not applicable	16 (5.5)	1 (0.8)	1 (0.9)	0.016
How do you cook your meat?				
Medium rare	70 (24.1)	21 (17.8)	14 (12.4)	0.025
Boiled	197 (67.7)	84 (71.2)	83 (73.5)	0.489
Grilled	158 (54.3)	51 (43.2)	28 (24.8)	< 0.001
Baked in the oven	175 (60.1)	54 (45.8)	41 (36.3)	< 0.001
Raw	3 (1)	1 (0.8)	4 (3.5)	0.144
Which of these do you consume?				
Raw milk	108 (37.1)	38 (32.2)	32 (28.3)	0.218
Testicle	47 (16.2)	1 (0.8)	8 (7.1)	< 0.001
Female genitals	0 (0)	2 (1.7)	0 (0)	0.032
Uncooked liver	99 (34)	25 (21.2)	11 (9.7)	< 0.001
Uncooked intestines	16 (5.5)	12 (10.2)	1 (0.9)	0.009
None of these	129 (44.3)	58 (49.2)	73 (64.6)	0.001
Raw milk is as healthy as supermarket milk	74 (25.4)	60 (50.8)	45 (39.8)	< 0.001
Should raw milk be boiled?	204(70.1)	84(71.2)	104(92.0)	< 0.001
Do you make homemade cheese?	10 (3.4)	4 (3.4)	7 (6.2)	0.414
Breeding methods				
Not applicable	179 (61.5)	17 (14.4)	19 (16.8)	< 0.001
I have males	103 (35.4)	99 (83.9)	94 (83.2)	< 0.001
I rent males	8 (2.7)	1 (0.8)	0 (0)	< 0.00
Artificial insemination	1 (0.3)	1 (0.8)	0 (0)	< 0.00
Do you need more information on brucellosis?	146 (50.2)	53 (44.9)	62 (54.9)	0.317
Which source of information do you prefer?				
MOH personnel	68 (23.4)	15 (12.7)	19 (16.8)	0.034
Video	39 (13.4)	11 (9.3)	2 (1.8)	0.002
Text	34 (11.7)	26 (22.0)	26 (23.0)	0.004

Data are presented as *n* (%) unless otherwise specified. MOH, Ministry of Health.

### 3.5 Factors associated with good attitude and practice regarding brucellosis by farmers and animal owners

Although no factor was significantly associated with high odds of vaccinating the livestock against brucellosis, the age group 21–30 years were less likely to provide such vaccine to their animals (OR, 0.28; 95% CI, 0.09–0.87; *p* = 0.027). As far as the attitude of the participants toward sick animals and protecting themselves and their animals from brucellosis, we found that only farmers and those with middle school or no education would more likely take their sick animals to the veterinarian (OR, 2.34; 95% CI, 1.15–4.76; *p* = 0.019, OR, 3.03; 95% CI, 1.28–7.16; *p* = 0.012, and OR, 2.42; 95% CI, 1.05–5.58; *p* = 0.038, respectively). While participants from the southern region and those with 5–10 years of experience would most likely take an aborted fetus and placenta to the veterinarian (OR, 11.78; 95% CI, 3.71–37.41; *p* < 0.0001 and OR, 4.58; 95% CI, 2.02–10.41; *p* < 0.0001), commercial owners are less likely to do so (OR, 0.37; 95% CI, 0.17–0.84; *p* = 0.017). Avoiding raw milk consumption was significantly a common practice among participants from the northern region only (OR, 6.96; 95% CI, 2.51–19.31; *p* < 0.0001); though, no significant effect of the other factors, including being from any other region, was observed on this practice. Besides being from the central region, none of the other factors, including being from any other region, was significantly associated with the attitude of consuming cooked meat vs. rare or medium-rare cooked meat (OR, 6.73; 95% CI, 2.21–20.49; *p* < 0.001). Detailed results of the regression analyses of factors associated with different attitude and practice items are provided in Supplementary Table S3.

### 3.6 Knowledge and attitude of animal slaughterhouse workers regarding brucellosis

A total of 23 animal slaughterhouse workers were interviewed for the relevant questions in the survey. All the respondents were non-Saudis and had a median [interquartile range] age of 31 [22–38]. While more than half (52.2%; *n* = 12) had a work experience of <5 years, all the respondents (100%) have heard of brucellosis, mostly (*n* = 22; 95.7%) from a family member or a friend, followed by social media (*n* = 13; 56.5%). The majority of the respondents were aware that *Brucella* can infect humans (*n* = 22; 95.7%) and knew at least one correct symptom (*n* = 21; 91.3%) and one mode of transmission to humans (*n* = 22; 95.7%), as well as the fact that brucellosis is treatable in humans (*n* = 21; 91.3%). Conversely, none of the respondents knew how to be protected against the infection. While 69.6% (*n* = 16) claimed that they drink raw milk, 91.3% (*n* = 21) said that they consume uncooked liver and 78.3% (*n* = 18) consume raw intestines.

## 4 Discussion

This is the first study to assess the knowledge of brucellosis and attitude of people working with animals to manage and prevent the acquisition of this zoonotic infection in Saudi Arabia where it is endemic. Overall, it was noted that private owners, who mostly held a college degree (47.8%), had better knowledge and followed better practices compared with farmers who mostly lacked a school education (35.4%) or had below college education (60.6%).

Our study found that most participants have heard of brucellosis (91.4% of farmers, 95.8% of commercial owners, and 98.2% of private owners). These percentages exceeded those came from studies that assessed the awareness of the Saudi public of brucellosis, where four studies from different regions involving 311 to 1,244 participants (with a collective total of 3,353 participants) found that only more than half of the surveyed populations were aware of the disease (range 50–73.6%) (9–12). This shows that brucellosis is widely known in Saudi Arabia; though, people working with animals were more likely to have heard of it. Despite the high prevalence of brucellosis awareness in our study, a knowledge gap regarding its symptoms and transmission was observed, especially among those who lacked school education and those with short work experience (< 5 years). These results resemble findings from a similar study from Jordan (14). Meanwhile, the studies on the Saudi public revealed that 53.1–90% exhibited good knowledge of brucellosis, with gender, age, and education level influencing awareness (9–12). The significant association between higher educational levels and good knowledge of brucellosis reported in these studies was similar to our opposite finding that lack of education was associated with poor knowledge.

The clinical manifestations of brucellosis in humans can be nonspecific and may include fever as the major feature, night sweats, chills, arthralgia, weight loss, among others (3, 19, 20). In our study, the recognition of brucellosis symptoms in humans varied between the participants, where private owners (86.7%) and commercial owners (74.6%) demonstrated a better knowledge than farmers (45.9%) in recognizing fever as human brucellosis symptom. The variations became even more prominent regarding the recognition of arthralgia as a symptom. A good proportion of private owners (41.6%) and commercial owners (32.2%) identified it compared to farmers (26.8%). Furthermore, our study found a proportion of participants who were unsure about any symptoms (6.2% of private owners, 14.4% of commercial owners, and 29.9% of farmers). This suggests a potential knowledge gap regarding the full range of brucellosis symptoms in humans and underscores the importance of increasing general awareness about brucellosis and its potential impact on human health. In comparison, the Saudi public exhibited limited ability in recognizing fever as a major brucellosis symptom, where19.6–74.4% recognized it, whereas 1.8–63.5% recognized arthralgia (9–11). This suggests that the general public might have a basic understanding of some key symptoms, but targeted education for the general public and people working with livestock, particularly farmers, is crucial to ensure comprehensive awareness.

In animals, abortion is the major characteristic of brucellosis, followed by weak calves and decreased milk production (5). In our study, miscarriage was mostly identified as a major animal brucellosis symptom by private owners (61.9%) compared to commercial owners (27.9%) and farmers (6.5%), and the difference was statistically significant (*p* < 0.001). In contrast, a study from Jordan by Musallam et al. (14) found that 76.4% of livestock owners linked brucellosis to miscarriage. The disparity in the results between our study and the Jordanian study could be due to variations in the prevalence of brucellosis between livestock populations in Saudi Arabia and Jordan (6.7% in a sample from one region in Saudi Arabia vs. 38.7% in a sample from three regions in Jordan) (2, 21). Another possibility lies in the effectiveness of educational initiatives targeting people working with animals in each country.

Although vaccination of animals against brucellosis is the key to halt the disease from spreading to humans and other animals, human vaccines are not yet available. In cases where the animal vaccination is inaccessible, occupational hygiene would be crucial to protect oneself and the public from brucellosis. This includes washing of hands after being exposed to animals, such as after milking and slaughtering, as well as pasteurizing or boiling of dairy products before consuming them or selling them to the public (3, 19, 20). When assessed for safe practices to prevent brucellosis acquisition and transmission in our study, the responses varied among participants with 61.2% seeking veterinary assistance for sick animals, 36.4% adhering to safe disposal practices for aborted fetuses, 34% consuming raw milk. A similarity between our study and the study by Musallam et al. (14) from Jordan was seen with regards to the lack of safe practice of disposing of aborted fetuses (28.8% vs. 36.4% in our study) and with regards to raw milk consumption (26% vs. 34% in our study). In a cross-sectional study conducted in the Kafrelsheikh district of Egypt that focused on shepherds, a 20% seroprevalence of brucellosis in sheep was found (15). While shepherds displayed a good knowledge about brucellosis in their flocks, they lacked awareness of transmission routes to humans. Additionally, high-risk practices were identified, such as unsafe handling of parturition and disposal of aborted materials. The study recommended educational campaigns targeting shepherds. In contrast, a study done in rural Iran identified behavioral (such as boiling of raw milk and vaccination against brucellosis) and non-behavioral (such as age and educational level) factors influencing brucellosis prevention (16). The study underscored the importance of these factors in health education and promotion programs to address brucellosis effectively. Overall, these studies collectively highlight the importance of tailored educational interventions to address knowledge gaps and improve practices related to brucellosis in diverse populations.

In our study, we noticed that 41% of participants employ occupational hygiene practices as means of protection. Conversely, none of the participants in the Egyptian study reported utilizing any form of protective hygiene (15). Furthermore, the usage of gloves and masks was observed in less than 6% of respondents in the Jordanian study (14). Participants lacking knowledge regarding protection are the ones who expressed desire for additional information regarding brucellosis and strategies for safeguarding oneself against it. But overall, 51.2% of the participants in our study recognized the need for additional information on brucellosis. In the Jordanian study, most participants (93%) fell into the category of lacking knowledge about protective measures, where >32% of the participants exhibited a lack of understanding regarding protection and occupational hygiene (14). This observation suggests that the participants in Saudi Arabia possessed a greater level of knowledge concerning protection from brucellosis.

Testing of an animal presumed to have brucellosis is the first step to identify it. If positive, the infected animal should be isolated from the rest of the herd for approximately 30 days and then retested before returning it to the herd (5). Regarding protection of animals from brucellosis, the participants in our study were asked about measures to safeguard healthy animals from infected animals. Over 26% of the participants suggested isolating the infected animals from the healthy ones as a mean of animal protection. In comparison, the Jordanian study reported that only 10% of the participants advocated the isolation of the infected animal, while >55% agreed that authorities should sell the infected animals in the market (14). In the Egyptian study, none of the participants mentioned any protective measures, and > 80% stated that selling the infected animals in the regional market would be the appropriate course of action (15). Unfortunately, such inappropriate practice of selling infected animals combined with lack of auditing from the responsible authorities may contribute to the persistence of brucellosis in the countries where it is endemic.

Slaughterhouse workers (commonly known as abattoir workers) are equally prone to brucellosis, similar to livestock farmers and animal owners (3). Survey results from slaughterhouse workers in our study revealed a solid understanding of brucellosis and its zoonotic nature. Nevertheless, none of the participants were aware of protective measures to prevent contracting the disease. This was also evident in a study from Saudi Arabia by Almasri et al. (22) that surveyed a sample of 80 slaughterhouse workers on occupational practices during the busy season of Hajj (pilgrimage) that involves the sacrifice of over a million cattle. Surprisingly, most participants in that study (*n* = 74; 92.5%) admitted to never using disposable gloves during animal slaughter, and 58 (72.5%) did not employ face masks. However, 77 (96.2%) participants reported hand washing with water and soap after the slaughter process. While only six participants in the study by Almasri et al. (22) tested positive for *Brucella* IgM antibodies during the Hajj season (of a total 54 workers tested), only one complained of fever (38°C) and arthralgia. In a study conducted in Egypt, a similar trend of occupational malpractice related to glove usage was observed; though, participants demonstrated compliance with hand washing and the use of safety shoes and aprons (23). Interestingly, 75.2% (*n* = 173 of 230) of participants in that study tested positive for brucellosis using Rose Bengal test. Findings from our study as well as regional studies underscore the critical importance of educating slaughterhouse workers about their vulnerability to zoonotic infections, including brucellosis. Equipping them with knowledge about safe occupational practices is essential for safeguarding against this disease.

While this is the first study in Saudi Arabia that interviewed people working with livestock to evaluate their knowledge of brucellosis and their attitude towards it, a few limitations exist that should be acknowledged. One limitation is the reliance on self-reported responses, which may be susceptible to recall bias or social desirability bias, which could potentially impact the accuracy of the collected data as participants may not accurately remember certain information or may provide responses that align with societal expectations rather than their true beliefs or behaviors. Another limitation is the difficulty encountered in accessing and interviewing farmers in rural areas as only participants in large livestock markets were approached. Furthermore, the length of the questionnaire itself was another limitation. Its extensive nature has the potential to elicit respondent discomfort, which can negatively affect their willingness to fully engage with the survey and provide accurate responses.

## 5 Conclusion

This study revealed the need to augment the knowledge of people working with animals, particularly those with no school education and those with short work experience, to identify human and animal brucellosis symptoms and how to protect oneself and animals from this disease that is known to be endemic in Saudi Arabia. Overall, findings from our study and the previous similar studies from the countries around Saudi Arabia collectively emphasize the importance of tailored interventions (e.g., procurement of brucellosis vaccine and mandating its administration to all livestock animals), targeted educational campaigns, and collaborative efforts to address the multifaceted challenges of brucellosis control and prevention. Understanding the specific knowledge gaps and implementing context-specific strategies are crucial for effectively combating brucellosis and minimizing its impact on public health.

## Data availability statement

The raw data supporting the conclusions of this article will be made available by the authors, without undue reservation.

## Ethics statement

The studies involving humans were approved by the Research Ethics Committee of the Faculty of Pharmacy, King Abdulaziz University, Jeddah, Saudi Arabia (Ref. PH-1444-42). The studies were conducted in accordance with the local legislation and institutional requirements. The ethics committee/institutional review board waived the requirement of written informed consent for participation from the participants or the participants' legal guardians/next of kin because verbal consent to participate was obtained from each participant prior to the interview and survey completion.

## Author contributions

MoA: Data curation, Investigation, Writing – original draft. EA: Data curation, Investigation, Writing – original draft. AHA: Data curation, Investigation, Writing – original draft. ASA: Data curation, Investigation, Writing – original draft. OA: Data curation, Investigation, Writing – original draft. AAA: Data curation, Investigation, Writing – original draft. MaA: Supervision, Writing – review & editing. AT: Conceptualization, Formal analysis, Methodology, Resources, Supervision, Validation, Visualization, Writing – original draft, Writing – review & editing.
