# Supplementary material for: Assessment of knowledge, attitude, and practice related to brucellosis among livestock farmers and meat handlers in Saudi Arabia
Source: Front Vet Sci. 2024 Jun 24;11:1410330. doi: 10.3389/fvets.2024.1410330 (PMC11229521; doi:10.3389/fvets.2024.1410330)
Supplement: Supplementary file 1 [file Data_Sheet_1.PDF]

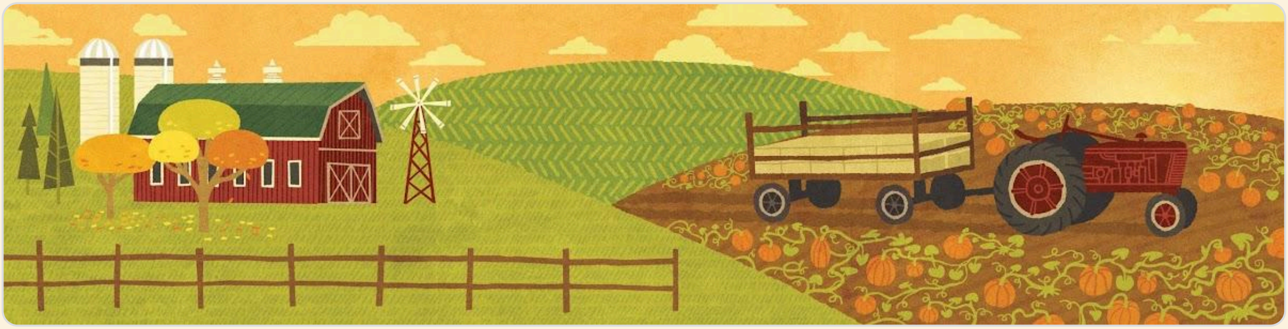

Brucellosis Survey among Farmers in Saudi Arabia

* Indicates required question

General Information

Age *

Your answer

Nationality *

- Saudi
- Sudanese
- Egyptian
- Yemeni
- Other:

City *

- Jeddah
- Taif
- Makkah
- Buraidah
- Unaizah
- Albukairiya
- Arras
- Albaha
- Taboos
- Abha/Khamis Mushait
- Medina
- Lady
- Other:

education level *

- No education
- Elementary
- secondary
- diploma
- University

Type of profession *

- ○ Shepherd
- ○ Commercial livestock owner
- ○ Personal livestock owner
- ○ Animal slaughterhouse
- ○ In the dairy products section

Number of years working with livestock *

Your answer

Have you heard about brucellosis? *

- ○ Yes
- ○ no

Where did you hear about brucellosis? *

- ☐ Friend or family
- ☐ Social media
- ☐ Television, radio or newspaper
- ☐ Health worker
- ☐ I haven't heard of it
- ☐ Other: ____________

Do you think you are at risk of contracting brucellosis? *

- ◯ Yes
- ◯ no

Which of the following symptoms did you experience last year? *

- ☐ Persistent fever
- ☐ Fever comes and goes
- ☐ Profuse sweating at night
- ☐ Exhaustion
- ☐ Arthritis
- ☐ Muscle pain
- ☐ headache
- ☐ Back ache
- ☐ nothing

Next

Clear form

Never submit passwords through Google Forms.

This form was created inside of King Abdulaziz University. Report Abuse

Google Forms

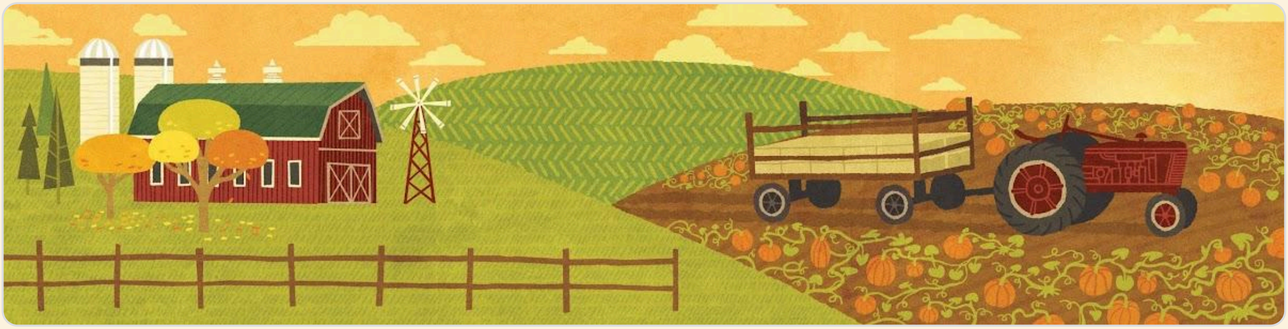

Brucellosis Survey among Farmers in Saudi Arabia

*Indications required question

Knowledge Knowledge

Can a person get brucellosis? *

- ◯ Yes
- ◯ no
- ◯ I don't know

How does a person get this disease? *

- [ ] Unpasteurized or sterilized dairy products
- [ ] Eat raw meat
- [ ] Inhaling polluted air when dealing with livestock
- [ ] Touching contaminated blood or slaughtered meat
- [ ] Touching livestock (while they are alive)
- [ ] I don't know
- [ ] Other: ____

How does brucellosis spread among livestock? *

Your answer

What are the symptoms of brucellosis in humans? *

Your answer

What are the signs of cattle infected with brucellosis? *

Your answer

Is brucellosis a treatable disease in humans? *

- ○ Yes
- ○ no
- ○ I don't know

How can brucellosis be prevented in humans? *

Your answer

How can brucellosis be prevented in livestock? *

Your answer

Back Next Clear form

Never submit passwords through Google Forms.

This form was created inside of King Abdulaziz University. Report Abuse

Google Forms

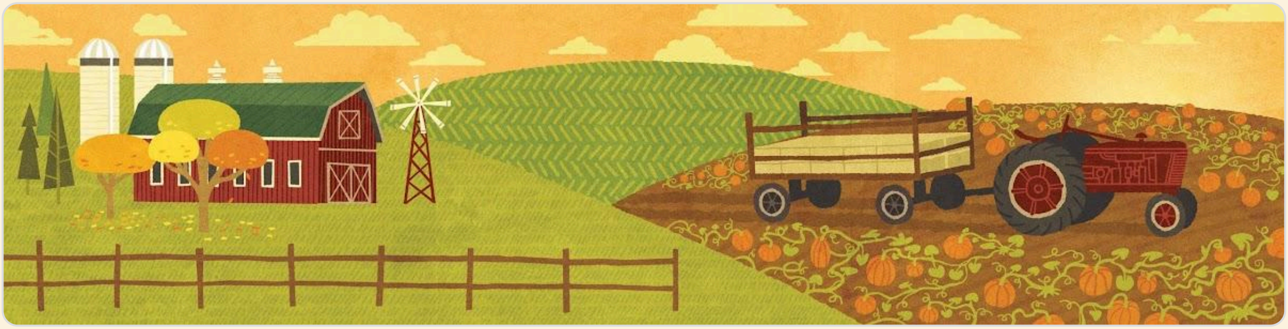

Brucellosis Survey among Farmers in Saudi Arabia

*Indications required question

Farmer Information

Number of cows *

Your answer

Number of camels *

Your answer

Number of sheep *

Your answer

Number of goats *

Your answer

How are cows raised on your farm? *

- ○ Cows share grazing with sheep
- ○ Cows share grazing with both sheep and goats
- ○ Cows are raised separately from sheep and goats
- ○ Only cows are raised, no goats and/or sheep
- ○ I don't have cows

Do cows share water with sheep or goats? *

- ○ Yes
- ○ no
- ○ do not apply

Source of drinking water for animals? *

- ○ Municipal water (from pipes from the National Water Company)
- ○ Desalinated water (white or bottled)
- ○ Well (groundwater)
- ○ do not apply
- ○ Other: ______

Livestock feed source? *

- From outside the farm
- From inside the farm
- do not apply

Where do you buy livestock for your farm? *

- From other livestock farms (or from land without an auction)
- From auctions
- From other countries
- No livestock are brought in from outside the farm
- do not apply
- Other: ____________________

Do you check the health of the animals before purchasing them? *

- No, I don't do any tests
- Yes, ask your vet to examine and perform tests
- Yes, I check the animals myself
- do not apply

How often do your cattle mix with cattle from other farms (eg at a livestock market)? *

- They always mix
- Sometimes they mix
- They don't mix at all
- do not apply

Where do you sell livestock from your farm? *

- In the livestock/cattle market
- At auctions
- From my personal farm
- At the local slaughterhouse
- Through export slaughterhouses (i.e. they are slaughtered and their meat is exported)
- Exported (while alive) to other countries
- do not apply

Do you sell animal milk (i.e. cow, goat, camel)? *

- Yes, on the farm
- Yes, off the farm
- We do not sell milk
- do not apply

What animal milk is consumed in your home ? *

- [ ] Cows
- [ ] Sheep
- [ ] Goat
- [ ] The Camels
- [ ] Milk is not consumed at home

The following products are made from fresh milk on the farm *

- [ ] the cheese
- [ ] Curd and/or yoghurt
- [ ] Ghee or butter
- [ ] nothing
- [ ] do not apply
- [ ] Other: ______

Who helps cattle give birth? *

Your answer ______

What vaccines do you give to livestock? *

- [ ] None (I do not vaccinate livestock)
- [ ] Universal vaccine
- [ ] do not apply
- [ ] Other: ____________

Back   Next   Clear form

Never submit passwords through Google Forms.

This form was created inside of King Abdulaziz University. Report Abuse

Google Forms

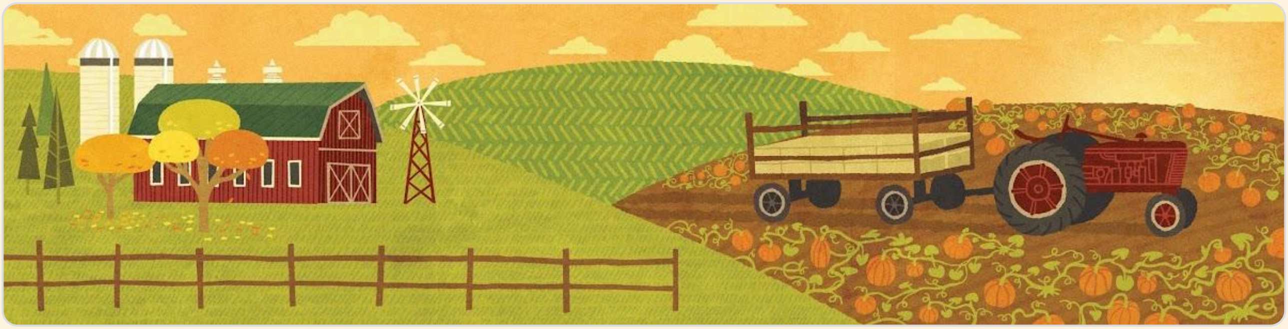

Brucellosis Survey among Farmers in Saudi Arabia

*Indications required question

Attitude

Do you vaccinate livestock against brucellosis? *

- ◯ Yes
- ◯ no
- ◯ do not apply

What vaccine do you use against brucellosis in cattle? *

Your answer

Did any of the livestock test positive for brucellosis last year? *

- Yes
- no
- do not apply

Who diagnosed the animal? *

If the answer above is “no,” write no

Your answer

What happened to the animals that tested positive for brucellosis? *

If the answer above is “no,” write no

Your answer

Is raw cow's (or other animal's) milk and homemade cheese as healthy as fresh packaged milk and supermarket cheese? *

- Yes
- no

Should raw milk (cow, goat, camel) be boiled before drinking it? *

- Yes
- no

How dangerous is abortion in livestock animals in your opinion? *

- Very serious
- serious
- not important

What do you do with livestock that get sick? *

- I treat it myself
- I'm looking for a vet
- I slaughter it to benefit from the meat
- do not apply
- Other: ____________

Do you need more information about brucellosis? *

- Yes
- no

How would you like to get information about brucellosis? *

If the answer above is "No" write No

Your answer

How do you milk animals (cows, goats, camels)? *

- By hand
- Through the milking machine
- We don't milk it
- Other: ____________________

Who milks the animals (cows, goats, camels)? *

If the answer above is "No" write No

Your answer ____________________

Do you wash your hands after milking animals (cows, goats, camels)? *

- Yes
- no
- I don't milk cows (not applicable)

Do you drink raw animal milk (cows, goats, camels)? *

- Yes
- no

Do you prepare and eat homemade (unpasteurized) cheese? *

- ◯ Yes
- ◯ no

Do you handle aborted fetuses and placentas with your hands? *

- ◯ Yes
- ◯ no

How do you deal with it? *

If the answer above is "No" write No

Your answer

What to do with aborted fetuses and placentas *

- ☐ Return it to the municipality (throw it on the street for the municipality to pick it up)
- ☐ Burn it
- ☐ Feed it to the dogs
- ☐ Bury her
- ☐ Call a vet
- ☐ Leave it without doing anything
- ☐ Other: ____________

What will you do if you suspect that an animal has brucellosis? *

Your answer

How do you prepare your meat when eating? *

- [ ] Raw
- [ ] Partially cooked
- [ ] grilled
- [ ] boiled
- [ ] in the Oven

Which of these things do you eat? *

- [ ] Raw milk
- [ ] Cattle testicles
- [ ] Uncooked liver
- [ ] The intestines are uncooked
- [ ] nothing
- [ ] Other: ____________

Enrichment methods used? *

- [ ] Artificial insemination
- [ ] I have males (example: bulls for cows, camels for camels, and goats for goats)
- [ ] I hire males
- [ ] do not apply
- [ ] Other: ____________________

Did you receive a circular from the Ministry of Agriculture requiring livestock to be vaccinated against brucellosis? *

- ( ) Yes
- ( ) no

Back | Submit | Clear form

Never submit passwords through Google Forms.

This form was created inside of King Abdulaziz University. Report Abuse

Google Forms
