# Supplementary material for: Assessment of knowledge, attitude, and practice related to brucellosis among livestock farmers and meat handlers in Saudi Arabia
Source: Front Vet Sci. 2024 Jun 24;11:1410330. doi: 10.3389/fvets.2024.1410330 (PMC11229521; doi:10.3389/fvets.2024.1410330)
Supplement: Supplementary file 2 [file Table_1.pdf]

Table S1. Data of animals and animal husbandry practices by farmers and animal owners in Saudi Arabia (n=522)

Item	Farmers (n=291)	Commercial Owners (n=118)	Private Owners (n=113)	*P* value
Number of cows (range)	0-22	0	0-1	-
Number of camels (range)	0-100	0-130	0-200	-
Number of sheep (range)	0-800	0-1000	0-2000	-
Number of goats (range)	0-300	0-200	0-350	-
Water source				< 0.001
Government	143 (49.1)	20 (16.9)	7 (6.2)	
Desalinated water	89 (30.6)	31 (26.3)	18 (15.9)	
Wells	59 (20.3)	67 (56.8)	88 (77.9)	
Fodder source				
The market	285 (97.9)	115 (97.5)	103 (91.2)	0.009
My farm	5 (1.7)	3 (2.5)	10 (8.8)	
Livestock source				< 0.001
Auctions	143 (49.1)	49 (41.5)	34 (30.1)	
Other farms	111 (38.1)	59 (50)	38 (33.6)	
Other countries	21 (7.2)	6 (5.1)	2 (1.8)	
Breeding	15 (5.2)	4 (3.4)	39 (34.5)	
Check livestock before purchasing				< 0.001
No	27 (9.3)	15 (12.7)	29 (25.7)	
Yes, by myself	213 (73.2)	89 (75.4)	59 (52.2)	
Yes, by vet	51 (17.5)	14 (11.9)	25 (22.1)	
Do you mix your livestock with animals from other farms?				< 0.001
Always	26 (8.9)	8 (6.8)	3 (2.7)	
Often	31 (10.7)	36 (30.5)	21 (18.8)	
Never	232 (79.7)	74 (62.7)	88 (78.6)	
Do you sell animal milk?				0.397
No	280 (96.2)	116 (98.3)	113 (100)	
Yes, inside the farm	6 (2.1)	1 (0.8)	0 (0)	
Yes, outside the farm	3 (1.0)	0 (0)	0 (0)	
Which of these products are made from fresh milk at your farm?				< 0.001
Yogurt	3 (1.0)	18 (15.3)	38 (33.6)	
Ghee/Butter	4 (1.4)	20 (16.9)	54 (47.8)	
Cheese	4 (1.4)	3 (2.5)	10 (8.8)	
None	282 (96.9)	97 (82.2)	54 (47.8)	
Who helps livestock to give birth?				< 0.001
None	68 (23.4)	15 (12.7)	25 (22.1)	
Grazer	166 (57.0)	80 (67.8)	77 (68.1)	
Veterinarian	12 (4.1)	6 (5.1)	4 (3.5)	
Owner	0 (0)	5 (4.2)	3 (2.7)	
I vaccinate my livestock	23 (17.9)	16 (13.6)	0 (0)	< 0.001
Vaccine type				
Total (Pasteurella & Clostridia)	92 (31.6)	72 (61)	87 (77.0)	< 0.001
Pasteurella only	2 (0.7)	0 (0)	0 (0)	
Clostridia only	3 (1)	0 (0)	1 (0.9)	0.549
Plague only	7 (2.4)	9 (7.6)	10 (8.8)	0.009
Chickenpox only	10 (3.4)	7 (5.9)	9 (8.0)	0.148
I don't vaccinate my animals	159 (54.6)	26 (22)	18 (15.9)	< 0.001

Data are presented as n (%) unless otherwise specified.

Table S2. Factors associated with good knowledge about brucellosis by farmers and animal owners in Saudi Arabia (n=522). Data are presented as odds ratios; 95% confidence intervals; *P* values

**Factors**	**Questions**							
	Brucella can infect humans	How is *Brucella* transmitted to humans?	How is *Brucella* transmitted between animals?	What are the symptoms of brucellosis in humans?	What are the symptoms of brucellosis in animals?	Brucellosis is treatable in humans	How can brucellosis be prevented in humans?	How can brucellosis be prevented in animals?
Age group (years)								
16–20	0.22; 0.02-2.78; 0.244	0.40; 0.06-2.52; 0.326	12.94; 1.99-84.20; 0.007	0.13; 0.01-1.39; 0.092	1.40; 0.21-9.23; 0.725	0.45; 0.02-8.83; 0.596	Not applicable[*]	4.29; 0.80-23.16; 0.090
21–30	0.79; 0.14-4.40; 0.788	0.50; 0.17-1.45; 0.201	2.17; 0.96-4.95; 0.064	0.89; 0.33-2.41; 0.823	1.57; 0.69-3.58; 0.284	9.02; 1.16-70.18; 0.036	0.59; 0.23-1.51; 0.269	1.45; 0.69-3.03; 0.324
31–50	1.35; 0.29-6.30; 0.702	1.07; 0.42-2.76; 0.890	2.57; 1.31-5.01; 0.006	1.18; 0.50-2.77; 0.703	1.73; 0.89-3.35; 0.107	3.96; 1.00-15.71; 0.05	0.37; 0.18-0.78; 0.009	1.82; 1.00-3.31; 0.049
> 50	Reference	Reference	Reference	Reference	Reference	Reference	Reference	Reference
Region								
Western	0.68; 0.23-2.03; 0.486	1.52; 0.64-3.61; 0.340	2.47; 1.12-5.51; 0.027	0.61; 0.26-1.45; 0.260	0.99; 0.45-2.17; 0.975	3.72; 0.71-19.52; 0.121	7.67; 0.86-68.56; 0.068	1.67; 0.80-3.48; 0.172
Southern	0.23; 0.06-0.89; 0.033	0.77; 0.28-2.13; 0.614	0.98; 0.37-2.60; 0.961	0.19; 0.07-0.54; 0.002	0.35; 0.13-0.93; 0.036	4.37; 0.62-30.86; 0.139	4.83; 0.52-45.28; 0.168	1.22; 0.51-2.93; 0.656
Central	5.14; 0.96-27.58; 0.056	1.23; 0.51-3.00; 0.643	2.81; 1.21-6.53; 0.016	0.84; 0.34-2.08; 0.706	1.61; 0.70-3.66; 0.261	68.21; 5.44-854.77; 0.001	7.68; 0.87-67.83; 0.067	2.01; 0.94-4.30; 0.072
Northern	0.19; 0.05-0.77; 0.020	0.77; 0.26-2.28; 0.637	1.69; 0.63-4.52; 0.294	0.36; 0.12-1.10; 0.073	0.82; 0.31-2.17; 0.690	1.32; 0.23-7.47; 0.756	15.14; 1.61-142.06; 0.017	0.98; 0.41-2.40; 0.966
Eastern	Reference	Reference	Reference	Reference	Reference	Reference	Reference	Reference
Education								
No education	0.39; 0.07-2.09; 0.273	0.30; 0.10-0.94; 0.038	0.13; 0.04-0.36; <0.0001	0.06; 0.02-0.23; <0.0001	0.13; 0.05-0.34; <0.0001	2.22; 0.36-13.57; 0.388	0.084; 0.03-0.25; <0.0001	0.90; 0.43-1.90; 0.790
Elementary	0.38; 0.07-1.97; 0.248	0.51; 0.16-1.67; 0.267	0.19; 0.07-0.57; 0.003	0.20; 0.05-0.76; 0.018	0.37; 0.13-1.02; 0.056	10.41; 0.86-125.60; 0.066	0.18; 0.07-0.50; 0.001	1.38; 0.64-3.01; 0.415
Middle School	0.31; 0.06-1.56; 0.154	0.45; 0.14-1.42; 0.172	0.15; 0.05-0.42; <0.0001	0.12; 0.03-0.44; 0.001	0.20; 0.07-0.53; 0.001	6.41; 0.88-46.92; 0.067	0.10; 0.04-0.27; <0.0001	1.22; 0.58-2.55; 0.604
High School	0.92; 0.17-4.98; 0.919	0.79; 0.26-2.47; 0.690	0.24; 0.08-0.65; 0.006	0.28; 0.08-0.10; 0.049	0.16; 0.06-0.42; <0.0001	1.96; 0.51-7.56; 0.330	0.33; 0.16-0.66; 0.002	1.71; 0.89-3.30; 0.108
Vocational College	0.33; 0.04-2.53; 0.284	0.74; 0.18-3.03; 0.670	0.25; 0.07-0.85; 0.027	0.43; 0.08-2.24; 0.314	0.28; 0.09-0.91; 0.034	0.96; 0.20-4.61; 0.957	0.73; 0.31-1.75; 0.482	1.01; 0.46-2.53; 0.866
College/University	Reference	Reference	Reference	Reference	Reference	Reference	Reference	Reference
Job								
Farmer	0.37; 0.09-1.51; 0.166	0.49; 0.18-1.37; 0.176	0.26; 0.18-0.89; 0.001	0.63; 0.25-1.56; 0.314	0.15; 0.07-0.33; <0.0001	3.00; 0.69-13.03; 0.144	0.26; 0.12-0.56; 0.001	2.09; 1.12-3.93; 0.022
Commercial owner	6.32; 0.99-40.47; 0.052	0.76; 0.26-2.16; 0.601	0.28; 0.17-0.85; 0.019	2.69; 0.93-7.79; 0.067	0.31; 0.14-0.67; 0.003	5.70; 1.17-27.77; 0.031	0.77; 0.39-1.49; 0.436	1.87; 1.02-3.46; 0.044
Private owner	Reference	Reference	Reference	Reference	Reference	Reference	Reference	Reference
Work experience (years)								

< 5	0.21; 0.04-1.12; 0.068	0.23; 0.08-0.62; 0.004	0.26; 0.11-0.58; 0.001	0.28; 0.11-0.72; 0.008	0.37; 0.16-0.82; 0.014	1.73; 0.25-11.91; 0.577	0.51; 0.20-1.28; 0.153	0.30; 0.15-0.60; 0.001
5–10	0.30; 0.06-1.51; 0.146	0.54; 0.21-1.40; 0.204	0.53; 0.27-1.06; 0.074	0.44; 0.19-1.02; 0.057	0.85; 0.43-1.66; 0.629	0.65; 0.16-2.67; 0.555	0.59; 0.28-1.26; 0.174	0.59; 0.32-1.07; 0.082
11–20	0.73; 0.14-3.75; 0.709	0.90; 0.34-2.43; 0.839	0.99; 0.50-1.98; 0.975	1.22; 0.51-2.92; 0.662	1.04; 0.54-2.02; 0.897	1.21; 0.32-4.63; 0.783	0.55; 0.26-1.16; 0.117	0.73; 0.41-1.31; 0.297
> 20	Reference	Reference	Reference	Reference	Reference	Reference	Reference	Reference

Note: Answering at least one correct answer indicated a good knowledge of the question.

* Due to having a value of zero in the number of participants aged 16-20 years who provided at least one correct answer to the question about the prevention of brucellosis in humans, this category was eliminated from the regression analysis to allow for appropriate computation of odds ratios for the other categories.

Table S3. Factors associated with good attitude and practice regarding brucellosis by farmers and animal owners in Saudi Arabia (n=522)

**Factors**	**Questions**				
	Vaccinate animals against *Brucella*	Take a sick animal to the veterinarian	Take an aborted fetus and placenta to the veterinarian	Avoid raw milk consumption	Consume cooked meat
Age group (years)					
16–20	Not applicable*	1.05; 0.10-10.84; 0.969	0.45; 0.06-3.24; 0.428	0.25; 0.04-1.66; 0.153	0.82; 0.05-14.86; 0.893
21–30	0.28; 0.09-0.87; 0.027	0.57; 0.24-1.34; 0.196	0.33; 0.13-0.84; 0.021	1.00; 0.46-2.16; 0.995	0.60; 0.18-1.97; 0.401
31–50	0.43; 0.19-1.00; 0.050	0.93; 0.47-1.86; 0.840	0.62; 0.29-1.33; 0.220	1.54;0.83-2.88; 0.174	0.92; 0.97-2.34; 0.868
> 50	Reference	Reference	Reference	Reference	Reference
Region					
Western	0.56; 0.13-2.46; 0.440	1.62; 0.73-3.60; 0.239	2.47; 0.86-7.07; 0.092	3.07; 1.46-6.48; 0.003	0.24; 0.06-0.99; 0.048
Southern	2.19; 0.51-9.40; 0.294	3.72; 1.39-9.98; 0.009	11.78; 3.71-37.41; <0.0001	1.88; 0.78-4.54; 0.163	0.29; 0.03-2.67; 0.273
Central	1.47; 0.36-6.00; 0.588	2.24; 0.96-5.20; 0.061	0.87; 0.28-2.67; 0.800	1.43; 0.67-3.04; 0.359	6.73; 2.21-20.49; 0.001
Northern	3.33; 0.74-15.13; 0.119	0.899; 0.35-2.32; 0.825	2.14; 0.63-7.31; 0.223	6.96; 2.51-19.31; <0.0001	0.29; 0.031-2.73; 0.278
Eastern	Reference	Reference	Reference	Reference	Reference
Education					
No education	0.34; 0.10-1.16; 0.085	2.42; 1.05-5.58; 0.038	1.51; 0.62-3.66; 0.364	0.48; 0.22-1.05; 0.066	2.20; 0.64-7.59; 0.212
Elementary	0.97; 0.32-2.95; 0.960	2.33; 0.97-5.58; 0.059	0.66; 0.25-1.74; 0.396	0.48; 0.21-1.10; 0.084	1.79; 0.49-6.52; 0.380
Middle School	0.34; 0.10-1.12; 0.075	3.03; 1.28-7.16; 0.012	0.96; 0.40-2.30; 0.926	1.07; 0.47-2.43; 0.877	0.14; 0.02-1.34; 0.088
High School	1.06; 0.44-2.56; 0.899	1.87; 0.92-3.77; 0.083	0.71; 0.32-1.59; 0.409	0.84; 0.41-1.71; 0.624	0.48; 0.27-2.61; 0.765
Vocational College	0.75; 0.22-2.58; 0.648	0.74; 0.31-1.78; 0.507	0.38; 0.12-1.23; 0.106	1.54; 0.54-4.40; 0.419	1.39; 0.36-5.41; 0.639
College/University	Reference	Reference	Reference	Reference	Reference
Job					
Farmer	0.53; 0.21-1.33; 0.174	2.34; 1.15-4.76; 0.019	0.48; 0.23-1.03; 0.058	1.02; 0.51-2.03; 0.953	2.41; 0.80-7.29; 0.120
Commercial owner	0.45; 0.20-1.05; 0.064	1.4; 0.72-2.72; 0.318	0.37; 0.17-0.84; 0.017	1.10; 0.57-2.14; 0.777	0.85; 0.30-2.39; 0.755
Private owner	Reference	Reference	Reference	Reference	Reference
Work experience (years)					
< 5	1.66; 0.59-4.65; 0.337	2.29; 1.03-5.12; 0.043	2.52; 0.99; 6.41; 0.052	1.86; 0.85-4.07; 0.120	2.22; 0.74-6.70; 0.156
5–10	0.80; 0.32-2.00; 0.626	1.84; 0.93-3.63; 0.081	4.58; 2.02-10.41; <0.0001	0.69; 0.36-1.31; 0.260	2.08; 0.79-5.46; 0.139
11–20	1.02; 0.42-2.47; 0.959	1.43; 0.74-2.74; 0.288	1.62; 0.70-3.71; 0.258	0.63; 0.34-1.16; 0.138	1.28; 0.49-3.33; 0.609
> 20	Reference	Reference	Reference	Reference	Reference

Note: Answering at least one correct answer indicated a good attitude and practice with regards to the question.
* Due to having a value of zero in the number of participants aged 16-20 years who provided at least one correct answer to the question about vaccination f livestock against brucellosis, this category was eliminated from the regression analysis to allow for appropriate computation of odds ratios for the other categories.
